# Down selecting adjuvanted vaccine formulations: a comparative method for harmonized evaluation

**DOI:** 10.1186/s12865-018-0245-0

**Published:** 2018-01-31

**Authors:** Sumera Y. Younis, Christophe Barnier-Quer, Simon Heuking, Vinod Sommandas, Livia Brunner, Nicole vd.Werff, Patrice Dubois, Martin Friede, Clemens Kocken, Nicolas Collin, Ed Remarque

**Affiliations:** 1 Biomedical Primate Research Centre, Department of Parasitology, Rijswijk, The Netherlands; 2 Vaccine Formulation Laboratory, University of Lausanne, Epalinges, Switzerland; 3 WHO, Geneva, Switzerland

**Keywords:** AMA1, *Plasmodium falciparum*, Hepatitis B, HBsAg, Tuberculosis, Ag85A, Adjuvants, Aluminium oxyhydroxide, Squalene-in-water SWE, QS21

## Abstract

**Background:**

The need for rapid and accurate comparison of panels of adjuvanted vaccine formulations and subsequent rational down selection, presents several challenges for modern vaccine development. Here we describe a method which may enable vaccine and adjuvant developers to compare antigen/adjuvant combinations in a harmonized fashion. Three reference antigens: *Plasmodium falciparum* apical membrane antigen 1 (AMA1), hepatitis B virus surface antigen (HBsAg), and Mycobacterium tuberculosis antigen 85A (Ag85A), were selected as model antigens and were each formulated with three adjuvants: aluminium oxyhydroxide, squalene-in-water emulsion, and a liposome formulation mixed with the purified saponin fraction QS21.

**Results:**

The nine antigen/adjuvant formulations were assessed for stability and immunogenicity in mice in order to provide benchmarks against which other formulations could be compared, in order to assist subsequent down selection of adjuvanted vaccines. Furthermore, mouse cellular immune responses were analyzed by measuring IFN-γ and IL-5 production in splenocytes by ELISPOT, and humoral responses were determined by antigen-specific ELISA, where levels of total IgG, IgG1, IgG2b and IgG2c in serum samples were determined.

**Conclusions:**

The reference antigens and adjuvants described in this study, which span a spectrum of immune responses, are of potential use as tools to act as points of reference in vaccine development studies. The harmonized methodology described herein may be used as a tool for adjuvant/antigen comparison studies.

## Background

Despite decades of important advances in vaccine research and development, effective vaccines for several infectious diseases, including AIDS/HIV, malaria and tuberculosis, have not yet reached the population at risk. Whilst modern antigens based on subunit/recombinant approaches are often more specific and generally safer, they also tend to be less immunogenic. Inadequate immunogenicity is one of the main reasons why vaccine developers turn to adjuvants, in the hope of improving levels and quality of the desired immune responses.

At present, the tools openly available that can assist in making an informed down selection following comparison of adjuvants and/or formulations are limited and not harmonized. Harmonization is important in order to have consistency when comparing adjuvants and/or formulations in studies. The development of harmonized assays, including harmonized reagents capable of providing benchmarking tools by which new adjuvants can be evaluated and down selected is of clear benefit. Such tools may also prove to be useful for the comparison of formulations across studies performed at different times and places. In order to develop such tools we have defined and established a set of laboratory assays and reagents that can be used as a harmonized benchmarking tool. This standardized approach will give researchers the opportunity to make informed decision when selecting specific adjuvants and/or formulations in their studies (down selection of candidates). These tools were developed using the following strategy: (i) surveying and analyzing the current down selection practices used in global adjuvant R&D, (ii) determining the assays that were the most appropriate for comparing adjuvant activities, (iii) disseminating the results and providing the necessary reagents to interested parties.

A written survey designed to collect information on adjuvant down selection practices was disseminated at the Modern Vaccines/Adjuvant Formulation (MVAF) Conference in Cannes, in October 2010, which was attended by stakeholders from industry and public research organizations. The survey was designed to capture an overview of the methods currently in use in adjuvant testing. The survey also included a number of questions on how a harmonized adjuvant evaluation method could function. The potential techniques identified in this survey led to the development of the adjuvant comparison tools described herein.

After analysis of the information collected, an adjuvant comparison assay was proposed, a set of in vivo experiments designed to evaluate the usefulness of the tool, and a study performed to investigate if the immune responses obtained could be used as a benchmark for other adjuvant comparison studies. As the in vivo methods which were to be used were critical, as were the reference materials and assays used in each of the experiments, it was decided to select three reference antigens and three reference adjuvants, to be used together with harmonized protocols in order to evaluate murine cellular (cytokine: Interferon-γ (IFN-γ) and interleukin-5 (IL-5) production in splenocytes) and humoral responses (antibodies: total IgG, IgG1, IgG2b and IgG2c in sera) induced by each formulation. In mice IFN-γ skews the antibody response towards the IgG2 isotype, whereas IL-5 skews towards an IgG1 response [1–3]. Assessment of the cellular and humoral responses is important to study the efficacy of a vaccine. In cellular responses T cells are activated which produce different cytokines. In our study we will study the production of IL-5, since this cytokine stimulates B cell growth and increases immunoglobulin secretion, which leads to higher humoral responses. Furthermore production of IFN-γ will be studied. This cytokine is critical for innate and adaptive immunity against viral, some bacterial, and protozoal infections. It activates macrophages and induces major histocompatibility complex (MHC) class II molecule expression in antigen presenting cells, which are important for initiating immune responses.

In humoral immune responses antibodies are produced by B cells which cause the destruction of extracellular microorganisms and prevent the spread of intracellular infections. In our study total IgG, IgG1, IgG2b and IgG2c in sera will be studied. IgG2a response in sera will not be studied, since the study will be conducted in C57BL/6 mice and mice with B6 background lack the Igh-1a allele that codes for IgG2a [4, 5]. Each of the antigens were chosen based on availability at GMP quality or as close to as possible, minimal intellectual property (IP) constraints, and model for parasitic, viral, and bacterial antigens. The following antigens were selected: (i) *Plasmodium falciparum* apical membrane antigen 1 (AMA1), a malaria vaccine candidate capable of inducing humoral responses [6, 7], (ii) hepatitis B viru*s* surface antigen (HBsAg), a particulate viral antigen that induces both cellular and humoral responses [8], and (iii) *Mycobacterium tuberculosis* antigen 85A (Ag85A), a tuberculosis vaccine candidate eliciting cellular responses [9, 10].

In addition, three reference adjuvants were selected based on availability at GMP quality or as close to as possible, distinct immunomodulatory properties, and distinct type of adjuvant class (delivery system vs. immunomodulator). The following adjuvants were selected: (i) aluminium oxyhydroxide (AlOH) as the most widely used class of adjuvant, and which induces mostly Th2-type skewed immune responses [11], (ii) a squalene-in-water emulsion (SWE) prepared at the Vaccine Formulation Laboratory, whose composition is similar to MF59™ (Novartis Vaccines and Diagnostics), an adjuvant extensively used in humans as part of seasonal and pandemic influenza [12], (iii) a liposomal formulation formulated with the purified saponin QS21 (QS21-Liposomes). QS21 is an adjuvant currently being tested in various clinical studies (including cancer, HIV and Alzheimer vaccines) and is also one component of the AS01 adjuvant system part of the Mosquirix™ malaria vaccine developed by GlaxoSmithKline Biologicals. QS21 has the ability to induce both B-cell and T-cell immune responses in a variety of preclinical models and in humans [13–15].

Each of the antigens was formulated separately with each of the adjuvants, resulting in a total of nine adjuvanted vaccine formulations. Antigens alone were not included in the in vivo study, as the main aim was to demonstrate the feasibility of an adjuvanted vaccine formulation tool.

## Methods

### Survey

An anonymous questionnaire was designed for research groups involved in vaccine development in industry and academia. The questionnaire was introduced to obtain an overview of the adjuvant testing methods currently in use in industry and academia. It also included a number of questions designed to gather information on a harmonized adjuvant evaluation method could be designed. Since there is no information in regard to patients’ confidentiality requested in this questionnaire, no ethical approval is needed (Official Journal of the European Union, L 119, 4 May 2016 [16]).

### Antigens and Adjuvants

AMA1 was produced at cGMP from FVO-strain [17, 18] and obtained from BPRC. GMP grade HBsAg was obtained from a non-disclosed Hepatitis B vaccine manufacturer. Ag85A was obtained from Lionex GmbH (Braunschweig, Germany).

AlOH was purchased from Brenntag (Ballerup, Denmark). Squalene-in-water emulsion SWE adjuvant was prepared by the Vaccine Formulation Laboratory (VFL) at the University of Lausanne, Epalinges, Switzerland. SWE comprises a metabolizeable oil (squalene 3.9% *w*/*v*), sorbitan trioleate (0.47% w/v), and polyoxyethylene (80) sorbitan monooleate (0.47% w/v) dispersed in 10 mM citrate buffer at pH 6.5. QS21 saponin was prepared by the VFL following purification of QuilA from *Quillaja saponaria Molina* (Brenntag, Denmark) [19]. Liposomes (10 mg/mL dioleoylphosphatidylcholine and 2.5 mg/mL cholesterol) were prepared at the VFL by dissolution of lipid and cholesterol in chloroform, rotary evaporation and rehydration of the lipid film with PBS and ultra-sonication followed by sterile filtration, as previously described [20].

### Preparation of formulations

The nine vaccine formulations were prepared under sterile conditions, in a biological safety cabinet.

Table 1 gives an overview of how the formulations were prepared. Of each formulation 500 μL was prepared. The same amount of each antigen (containing either 1 μg AMA1, 5 μg HBsAg or 10 μg Ag85A (final antigen dose)) was used in different adjuvant formulations. Per mouse 50 μL was injected at time of each vaccination.

**Table 1:** Preparation of the nine vaccine formulations (test group) and control groups

Formulations	Test group		Adjuvant-only control group
	Adjuvant suspension	Antigen solution	
AlOH	85 μL AlOH + 165 μL SFI	10 μg AMA1 in 250 μL SFI	250 μL adjuvant suspension + 250 μL PBS
		50 μg HBsAg in 250 μL SFI	
		100 μg Ag85A in 250 μL SFI	
HBsAg	250 μL SWE	40 μg/mL AMA1 in 250 μL PBS	250 μL adjuvant suspension + 250 μL PBS
		200 μg/mL HBsAg in 250 μL PBS	
		400 μg/mL Ag85A in 250 μL PBS	
Ag85A	100 μL QS21 (1 mg/mL) + 100 μL DOPC:Chol + 50 μL PBS	10 μg AMA1 in 250 μL PBS	200 μL adjuvant suspension + 300 μL PBS
		50 μg HBsAg in 250 μL PBS	
		100 μg Ag85A in 250 μL PBS	

AlOH formulations were prepared by mixing 85 μL of aluminium oxyhydroxide (containing 850 μg of elementary aluminium) with 165 μL of saline for injection (SFI) followed by vortexing at high speed for 5 s. 250 μL of antigen solution in SFI (containing either 10 μg AMA1, 50 μg HBsAg or 100 μg Ag85A) were mixed with AlOH suspension, followed by vortexing at high speed for 5 s. Adjuvant-only control was prepared by mixing 250 μL of SFI with 250 μL of AlOH suspension (control group).

SWE formulations were prepared by mixing 250 μL of SWE with 250 μL of antigen solution in PBS (containing either 40 μg/mL AMA1, 200 μg/mL HBsAg or 400 μg/mL Ag85A), followed by vortexing at high speed for 5 s. Adjuvant-only control was prepared by mixing 250 μL of PBS with 250 μL of SWE (control group).

QS21-Liposome formulations were prepared by adding 100 μL of QS21 solution in PBS (at 1 mg/mL) to 100 μL of DOPC:Chol liposome suspension, followed by gentle inversion and addition of 50 μL of PBS. After 5 min QS21-Liposomes suspension was mixed with, 250 μL of antigen solution in PBS (containing either 10 μg AMA1, 50 μg HBsAg or 100 μg Ag85A), followed by gentle inversion. The adjuvant-only control (so no antigen) was prepared by mixing 300 μL of PBS to 200 μL of QS21-Liposome suspension (control group).

All formulations were injected within 2 h of preparation.

### Characterization of formulations

Following preparation, a compatibility and stability study of the nine different antigen-adjuvant combinations was performed over a 24 h period of storage at room temperature (RT), RT in the testing laboratory was between 18 and 22 °C). The characterization methods used were selected in accordance with each antigen and adjuvant. AlOH formulations were first analyzed visually for potential flocculation and centrifuged at 16,000 g for 2 min at RT. After centrifugation, 400 μL of supernatant was quantified for non-adsorbed antigen, either by ELISA (AMA1 and HBsAg formulations) or for Ag85A formulations by Micro BCA (Thermo Scientific, Wohlen, Switzerland). The antigen adsorption rates were extrapolated from the non-adsorbed antigen results.

SWE formulations and QS21-Liposome formulations were characterized for particle size and/or zeta potential using ZetaSizer® Nano ZS (Malvern, United Kingdom). Samples were analyzed in triplicates at 20 °C. Antigen integrity was analyzed by silver-stained SDS-PAGE (Invitrogen, Lucerne, Switzerland).

### Mouse immunization and blood sampling

All animal work was performed under the guidelines of Biomedical Primate Research Centre (BPRC) which uses protocols conforming to European animal welfare regulations. The independent ethics committee at BPRC, constituted according to Dutch law on animal experiments, approved the study protocol (number DEC 658) prior to start of the experiment. Immunization studies were carried out in nine groups of nine female C57BL/6 mice (test groups) and three groups of three female C57BL/6 mice (control groups).

Each animal was immunized (50 μL) intramuscularly (i.m.) with AMA1, HBsAg or Ag85A antigens, combined to one of the three adjuvants tested in this study. Immunizations were performed under isoflurane inhalation anesthesia. Control groups, one for each adjuvant, were immunized with adjuvant only as described in paragraph *Preparation of formulations* and Table 1. Control vaccines were administered at four weekly intervals (days 0, 28 and 56). Blood samples (100 μL) were taken at days 0 and 42. Animals were euthanized by cervical dislocation under isoflurane at week 10 (day 70) when blood and spleens were collected for analysis. The in vivo experiment was performed only once. Seven mice were lost due to experimental procedures (anesthesia) not related to treatment. AMA1: AlOH/QS21 2 mice, HBsAg: SWE 1 mouse, Ag85A: QS21 2 mice, and SWE 2 mice.

### ELISPOT

IFN-γ and IL-5 ELISPOT analyses were performed with splenocytes obtained on day 70. Splenocytes were obtained by passing mashed spleens through a 70 μm cell strainer to a single cell suspension. Cells were washed twice with RPMI and adjusted to a final volume of 5 mL in culture medium (CM). CM contains RPMI (Gibco, Invitrogen, Breda, The Netherlands) + 10% filtered FCS (Gibco, Invitrogen, Breda, The Netherlands) + 1% Glutamax (Gibco, Invitrogen, Breda, The Netherlands) + 1% Penicillin/Streptomycin (Gibco, Invitrogen, Breda, The Netherlands). Counting of cells was done using Casy Counter (Cell Counter and Analyser System - Model TT, Schärfe System – Reutlingen, Germany). Cells were plated at 5 × 10^5^ cells per well and stimulated overnight with 10 μg/mL antigen (AMA1, HBsAg or Ag85A). PMA-Iono (1 μg/mL) was included as a positive control (U-CyTech, Utrecht, The Netherlands). All samples were tested in triplicate.

ELISPOT plates (MultiScreen HTS and Hi-flow, MilliPore, Amsterdam, The Netherlands) were coated overnight with anti-cytokine antibodies – IFN-γ and IL-5 (U-CyTech kit, Utrecht, The Netherlands) according to manufacturers’ instructions. Following 24 h the stimulated splenocytes were transferred into the coated ELISPOT plates and incubated for 24 h at 37 °C in a 5% CO_2_ gassed incubator. ELISPOT plates were developed according to manufacturers’ instructions using Streptavidin-HRP conjugate (U-CyTech, Utrecht, The Netherlands). Spots were counted using AELVIS (Elispot Reader, Hannover, Germany). ELISPOT counts are presented as medium corrected counts per 10^6^ cells (i.e. counts obtained with medium only are subtracted from the counts obtained with antigens). In the event medium stimulated cells had higher counts than antigen stimulated cells 0 spot counts were arbitrarily assigned.

### Enzyme-linked immunosorbent assay

HBsAg ELISA kit was obtained from Alpha Diagnostic (San Antonio, USA). AMA1 ELISA (including 4G2 rat antibody, BG98, goat anti rabbit IgG conjugated to alkaline phosphatase, para-nitro phenol phosphate and diethanolamine) was provided by BPRC and was used as described previously [6]. ELISA was performed on serum samples in 96-well flat bottom Microlon titre plates (Greiner, Alphen a/d Rijn, The Netherlands). Plates were coated with 1 μg/mL antigen (AMA1 or HBsAg or Ag85A antigen (100 μL/well)) at 4°C overnight. After blocking with 200 μL/well of 3% BSA (Sigma, Zwijndrecht, The Netherlands) in PBS-T, day 0 – day 42 – day 70 serum samples were loaded to the plates and incubated for 1 h at RT. Mouse day 0 samples were tested at 1:100 and 1:500 for IgG-total and day 42 – day 70 samples at different dilutions for the four IgG subclasses: total IgG – IgG1 – IgG2b – IgG2c. All samples were tested in duplicate.

For mouse ELISA a pool of day 70 sera samples was used as a standard on every plate in a 2-fold serial dilution series. After sample incubation, plates were developed with 100 μL/well of 1:1000 diluted goat anti-mouse antibody conjugated to horseradish peroxidase for all four subclasses (Invitrogen, Breda, The Netherlands). ELISA development was with 100 μL/well ready-to-use TMB-substrate (Kem-en-Tec, Taastrup, Denmark) (for 15–20 min and stopped with 50 μL/well 2 mM H_2_SO_4_. The optical density (OD) was read at 450 nm using BioRad platereader (Model iMark – microplate reader, Bio-Rad, Veenendaal, The Netherlands).

ODs were converted to arbitrary units (AUs) using a four-parameter-logistic-fit (ADAMSEL, www.malariaresearch.eu), were 1 AU yields an OD of 1 over background. Thus the amount of AU of a sample is the reciprocal dilution at which an OD of 1 over background will be achieved. A standard curve was included on every plate.

### Statistical analysis

All data are presented as medians with 25 and 75% percentiles. Statistical significance was assessed by non-parametric tests; a *p*-value < 0.05 is considered statistically significant. Differences in antibody levels between the three adjuvant groups were initially compared using the Kruskal-Wallis (KW) test, where a *p*-value smaller than 0.05 indicates that at least two of the three groups differ significantly. In the event of a significant KW *p*-value, differences between the three adjuvant groups were subsequently evaluated by Mann-Whitney U (MW) tests. As three comparisons can be made, the p-value at which significance is considered requires adjustment by the Bonferroni method, i.e. *p*-values smaller than 0.05/3 = 0.0167 were considered statistically significant for the (secondary) Mann-Whitney comparisons. Therefore the p-value was set at 0.0167 as significance level for secondary comparisons.

## Results

### Survey and outcomes

In total 12 surveys were returned; six from research groups in industry and six from research groups in academia and/or governmental institutions. From the completed questionnaires, it was evident than no two research groups used similar methods for adjuvant comparison when down selecting candidates for their research. Evaluation of adjuvant activity was mainly done in mice, which was one of the few consensus points. A plethora of mice strains, immunization schedules, dosages and routes of immunization were listed. Based on the questionnaire responses and an analysis of the available literature it was clear that no ‘best practice’ evaluation method was prevalent, and that there was a clear need for a method and protocol to be chosen as the basis for a harmonized method.

As a first step, a harmonized animal model was selected. The questionnaire revealed that two mouse lines, BALB/c and C57BL/6 mice, were most commonly used. As there are several issues associated with the BALB/c mouse that could bias adjuvant comparisons, such as IL-12 receptor insensitivity and sub-lines from different suppliers, [9, 21] it was therefore decided to use C57BL/6 mice.

Next, a route model was selected. The i.m. route of immunization was chosen as the questionnaire responses revealed a preference for this route, and it also is the most frequently used route for human vaccine administration. Nevertheless, the immunization volumes are 50 μL, which is a small volume which can easily be injected in the quadriceps of the mice [22].

Finally, a schedule model was selected. Three injections were most commonly mentioned in the questionnaire results although there was no consensus on the timing of these immunizations. Immunizations on days 0, 28 and 56 (which is similar to schedule of the World Health Organization (WHO) Expanded Programme on Immunisations (EPI)) was selected.

### Characterization and stability of vaccine formulations

The different types of adjuvants used in the study have specific characterization tools. For aluminium salts, high importance is given to the antigen adsorption. It is usually recommended that at least 80% of the vaccine antigens be adsorbed on aluminium, [23]. In our study, all the supernatants tested were below the level of antigen detection (ELISA limit of quantification was 5 ng/mL for HBsAg and 12.5 ng/mL for AMA1, and on mBCA™ limit of quantification was 2 μg/mL) which has demonstrated a high degree of antigen adsorption exceeding 95% for all the antigen-aluminium formulations and remained constant after 24 h at RT (Table 2). Besides, none of the AlOH formulations showed any signs of flocculation suggesting physico-chemical instability.

**Table 2:** Characterization of formulations developed for the study. Showing adsorption percentages after 2 h and 24 h for AlOH formulations; Zeta potential and pH for SWE formulations; particle size and pH for QS21-Liposome

	AlOH		SWE		QS21-Liposomes	
Antigen	Time (h)	Adsorption (%)	Size (nm)	pH	Size (nm)	pH
AMA1	2	> 99.9^a^	133.8 ± 1.2	6.27	130.8 ± 0.4	6.88
	24	> 99.9^a^	133.9 ± 1.0	6.29	121.2 ± 1.6	6.98
HBsAg	2	> 99.9^a^	133.5 ± 1.5	6.26	134.0 ± 1.3	7.18
	24	> 99.9^a^	133.6 ± 1.1	6.26	126.7 ± 0.1	7.14
Ag85A	2	> 98.0^b^	133.2 ± 1.1	6.48	139.6 ± 3.9	7.31
	24	> 98.0^b^	134.3 ± 0.9	6.53	138.9 ± 1.2	7.37
Control	2	N/A	134.4 ± 2.3	6.50	132.1 ± 2.6	7.36
	24	N/A	134.3 ± 1.0	6.51	111.7 ± 12.0	7.36

^a^based on ELISA results (limit of quantification was 12.5 ng/mL for AMA1 and 5 ng/mL for HBsAg)

^b^based on mBCA™ (limit of quantification was 2 μg/mL)

In the case of SWE emulsion and liposomes, the particle size stability during storage is one of the criteria of importance. Hydrodynamic diameter of SWE particles remained constant at ~ 134 nm. Regarding QS21-Liposomes formulations, the hydrodynamic diameter remained in the 110–130 nm range. For all formulations, the integrity for all antigens was confirmed in comparison to controls by SDS-PAGE and/or ELISA (data not shown) and after 24 h storage.

### ELISPOT / IFN-γ

**Fig. 1:**
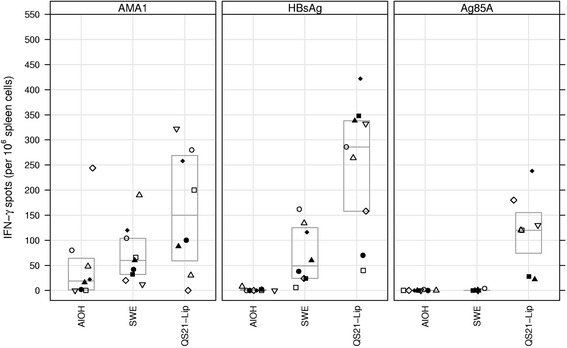
IFN-γ spots per 10^6^ spleen cells plotted for the three antigens with the three adjuvants. Boxes indicate quartile ranges (bottom and top) and medians (middle). Same symbol within each treatment group refers to the same animal throughout all graphs

No IFN-γ responses exceeding background were observed in the control mice that received adjuvant only upon antigen stimulation (data not shown). For AMA1 the median number of IFN-γ spots showed an increasing trend over the adjuvants/groups from 19 in the AlOH, to 60 for the SWE and to 150 for the QS21-Liposomes groups (Fig. 1), but none of the comparisons reached statistical significance (*p* = 0.081, KW test). For HBsAg, statistically significant differences were observed between the adjuvant groups (*p* < 0.00005, KW test), with AlOH, SWE and QS21-Liposomes yielding medians of 0, 49 and 286 spots, respectively (Fig. 1) and all adjuvant comparisons reached statistical significance (all *p* < 0.001, MW test). For Ag85A, statistically significant differences were observed between the adjuvant groups (*p* = 0.00012, KW test); the AlOH and SWE groups did not show IFN-γ responses exceeding background values (median count 0), whereas all animals in the QS21-Liposomes group showed responses exceeding background and as a group had a median count of 120 spots (p = 0.0001 KW test and *p* < 0.002 for comparisons of QS21-Liposomes with AlOH or SWE MW test) (Fig. 1).

### ELISPOT - Il-5

**Fig. 2:**
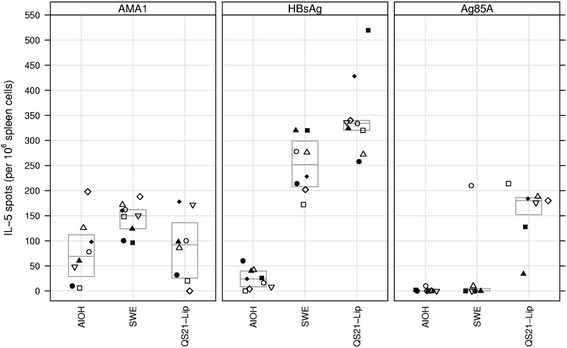
IL-5 spots per 10^6^ spleen cells plotted for the three antigens with the three adjuvants. Boxes indicate quartile ranges (bottom and top) and medians (middle). Same symbol within each treatment group refers to the same animal throughout all graphs

No IL-5 responses exceeding background were observed in the control mice that received adjuvant only upon antigen stimulation (data not shown). For AMA1 the median number of IL-5 spots was 69, 150 and 92 for AlOH, SWE and QS21-Liposomes respectively, this, however, failed to reach statistical significance (*p* = 0.064, KW test) (Fig. 2). For HBsAg statistically significant differences were observed in the number of IL-5 spots, with median spot counts of 24, 252 and 334, for AlOH, SWE and QS21-Liposomes, respectively (*p* < 0.00005, KW test and all between groups comparisons *p* < 0.011, MW test) (Fig. 2). For HBsAg, the number of IL-5 spots was significantly higher in the QS21-Liposomes group as compared to the AlOH group (all *p* < 0.001, MW test) (Fig. 2). The number of IL-5 spots tended to be higher in the QS21-Liposomes group as compared to the SWE-group, but this did not reach statistical significance (*p* = 0.019, MW test). For antigen 85A both AlOH and SWE failed to induce significant IL-5 responses, with only one animal in the SWE group clearly responding. By contrast, all animals in the QS21-Liposomes group had a detectable IL-5 response with a median count of 180 spots (Fig. 2).

### Antibody levels at baseline

Pre-vaccination total IgG serum titres were determined in day 0 serum samples. All animals were negative for total IgG to the antigen under investigation (data not shown) and no significant differences were observed between the treatment groups for each antigen (*p* = 0.4573 for AMA1; *p* = 0.1893 for HBsAg and *p* = 0.3393 for Ag85A, KW test).

### Total IgG

**Fig. 3:**
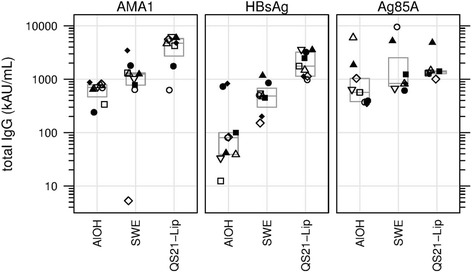
IgGt Median number of total IgG titres in AU/mL shown for the three antigens with the three adjuvants. Same symbol within each treatment group refers to the same animal throughout all graphs

At day 70 median IgG titres to AMA1 showed an increasing trend over the adjuvant groups, with AlOH yielding lowest, SWE intermediate and QS21-Liposomes yielding highest responses (*p* = 0.004, KW test), although non responders were also observed. Day 70 titres were significantly higher in the QS21-Liposomes group as compared to the AlOH and SWE groups (*p* = 0.0047 and 0.0111, respectively, MW test) (Fig. 3). Day 70 median IgG titres to HBsAg showed an increasing trend over the adjuvant groups, with AlOH yielding lowest, SWE intermediate and QS21-Liposomes yielding highest responses (*p* < 0.0001, KW test). Day 70 titres were significantly higher in the QS21-Liposomes group as compared to the AlOH and SWE groups (p < 0.0001 and < 0.001, respectively, MW test) (Fig. 3). At day 70 no statistically significant difference were observed in IgG titres to Ag85A for the three adjuvant groups (*p* = 0.0818, KW test) (Fig. 3).

### IgG1

**Fig. 4:**
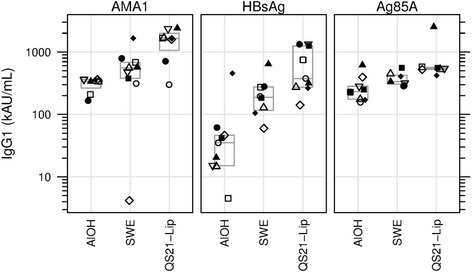
IgG1 Median number of IgG1 titres in AU/mL shown for the three antigens with the three adjuvants. Same symbol within each treatment group refers to the same animal throughout all graphs

At day 70 median IgG1 titres to AMA1 showed an increasing trend over the adjuvant groups, with AlOH yielding lowest, SWE intermediate and QS21-Liposomes yielding highest responses (p = 0.004, KW test). Day 70 IgG1 titres differed significantly for the AlOH and QS21-Liposomes groups (p = 0.0047, KW test) (Fig. 4). Day 70 median IgG titres to HBsAg showed an increasing trend over the adjuvant groups, with AlOH yielding lowest, SWE intermediate and QS21-Liposomes yielding highest responses (*p* = 0.0007, KW test). Day 70 titres were significantly higher in the SWE and QS21-Liposomes groups as compared to the AlOH group (*p* = 0.0055 and 0.0008, respectively, MW test) (Fig. 4). At day 70 statistically significant differences were observed in IgG1 titres to Ag85A for the three adjuvant groups (*p* = 0.0038, KW test). IgG1 titres were higher in the SWE group as compared to the AlOH group (*p* = 0.0311, MW test), but this was not statistically significant after correction for multiple tests. Day 70 IgG1 titres were highest in the QS21-Liposomes group and this was significantly higher as compared to the AlOH group (*p* = 0.0052, MW test), but failed to reach significance for the comparison with the SWE group after correction for multiple tests (*p* = 0.0175, MW test) (Fig. 4).

### IgG2b

**Fig. 5:**
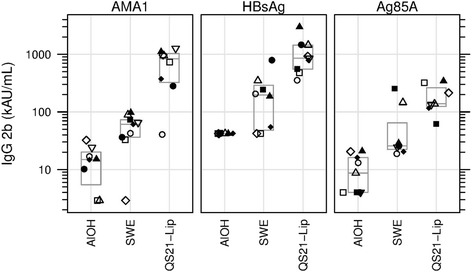
IgG2b Median number of IgG2b titres in AU/mL shown for the three antigens with the three adjuvants. Same symbol within each treatment group refers to the same animal throughout all graphs

At day 70 median IgG titres to AMA1 showed an increasing trend over the adjuvant groups, with AlOH yielding lowest, SWE intermediate and QS21-Liposomes yielding highest responses (*p* = 0.0002, KW test). Day 70 titres were significantly higher in the SWE and QS21-Liposomes groups as compared to the AlOH group (*p* = 0.0060 and 0.0009, respectively, MW test) and QS21-Liposomes group compared to SWE group (*p* = 0.0025) (Fig. 5). Day 70 median IgG2b titres to HBsAg showed an increasing trend over the adjuvant groups, with AlOH yielding lowest, SWE intermediate and QS21-Liposomes yielding highest responses (*p* = 0.0001, KW test). Day 70 titres were significantly higher in the SWE and QS21-Liposomes groups as compared to the AlOH group (Fig. 5, p = 0.0060 and 0.0002, respectively, MW test) and QS21-Liposomes group compared to SWE group (*p* = 0.0024) (Fig. 5). At day 70 statistically significant differences were observed in IgG2b titres to Ag85A for the three adjuvant groups (Fig. 5, *p* = 0.0004, KW test). IgG2b titres were higher in the QS21-Liposomes group as compared to the AlOH group and higher in the SWE group compared to the AlOH group (*p* = 0.0009 and *p* = 0.0040, respectively, MW test), these results were statistically significant. However the QS21-Liposomes group IgG2b titres compared to the SWE group failed to reach statistical significance after correction for multiple tests (Fig. 5, *p* = 0.0530, MW test).

### IgG2c

**Fig. 6:**
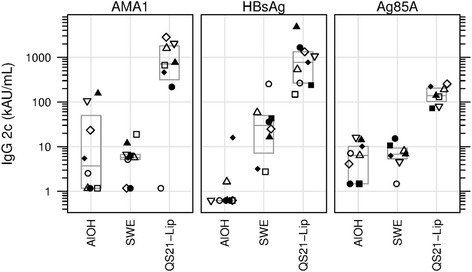
IgG2c Median number of IgG2c titres in AU/mL shown for the three antigens with the three adjuvants. Same symbol within each treatment group refers to the same animal throughout all graphs

At day 70 median IgG2c titres to AMA1 showed an increasing trend over the adjuvant groups, with AlOH yielding lowest, SWE intermediate and QS21-Liposomes yielding highest responses (Fig. 6, *p* = 0.0072, KW test). Day 70 titres were significantly higher in the QS21-Liposomes group as compared to the AlOH and SWE groups (*p* = 0.0082 and 0.0080, respectively, MW test) (Fig. 6). At day 70 median IgG2c titres to HBsAg showed an increasing trend over the adjuvant groups, with AlOH yielding lowest, SWE intermediate and QS21-Liposomes yielding highest responses (Fig. 6, *p* = 0.0000, KW test). Day 70 titres were significantly higher in the SWE and the QS21-Liposomes groups as compared to the AlOH group (*p* = 0.0008 and 0.0003, respectively, MW test) and QS21-Liposomes group compared to SWE group (Fig. 6, *p* = 0.0003). At day 70 statistically significant differences were observed in IgG2c titres to Ag85A for the three adjuvant groups (Fig. 6, p = 0.0008, KW test). IgG2c titres were higher in the QS21-Liposomes group as compared to the AlOH group and the SWE group (*p* = 0.0010 and 0.0006, respectively, MW test), these results were statistically significant. However, IgG2c titres of the SWE group compared to the AlOH group failed to reach statistical significance after correction for multiple tests (*p* = 0.7090, MW test) (Fig. 6).

Overall QS21-Liposomes group yielded highest IgG levels for all four IgG subtypes for all three antigens. SWE group yielded in intermediate IgG levels for all four IgG subtypes for all three antigens. AlOH group yielded in low IgG levels for all four IgG subtypes for AMA1 and HBsAg. For Ag85A the AlOH group yielded in intermediate levels for IgG1, IgG2b, and IgG2c. Total IgG levels for Ag85A were overall the same.

## Discussion

The findings described above have allowed the development of a harmonized protocol which may prove useful for the down selection of vaccine adjuvants during preclinical studies. The data presented here is the foundation of this harmonization, and compares the immune responses observed in mice using three adjuvants formulated with three model antigens. The data presented here show that QS21 is an adjuvant capable of inducing IFN-γ resulting in an IgG2 dominated IgG response, whereas AlOH and SWE tend to induce an IL-5 dominated cellular- and IgG1 dominated IgG response.

The immunological outcome of the in vivo study revealed different immunological effects induced by the different antigens combined with the same adjuvant. The QS21-Liposome formulations (in combination with all three antigens) induced a strong IFN-γ response, which was confirmed by the level of IgG2b and IgG2c as detected by ELISA. In contrast, no IFN-γ production was detected when AlOH was combined with HBsAg and Ag85A. This result was expected as AlOH is known to be a weak inducer of IFN-γ type-responses [24]. The SWE-adjuvanted group did not show any production of IFN-γ when in combination with Ag85A, whereas some IFN-γ responses were observed in combination with AMA-1 and HBsAg, alongside an increase in the IgG2b ELISA titers. The level of Th-2 cytokines, represented here by IL-5, showed that the QS21-Liposome adjuvant was a potent inducer of IL-5 when combined with all the three antigens. However, when combined with AlOH, only HBsAg and AMA1 immunization elicited low numbers of spots whereas superior levels of IL-5 secretion were detected for these two antigens when formulated with SWE. Moreover, production of IFN-γ is known to induce B cells to bias the production of IgG2b and IgG2c subclasses, whereas the production of IL-5 biases B cells to produce more total IgG and IgG1 [25]. The data presented in this paper are in accordance with this previous work.

The differences observed in the number of IFN-γ and IL-5 spots in this study could potentially be a result of the different concentrations used for the three antigens (the selected concentrations were based on measurable responses in previous studies) [6, 26, 27] or the different T-cell re-stimulation conditions used. Therefore, from these results, it is still difficult to compare the intensity of immune responses obtained with the different antigens, and it may be more beneficial to focus on the quality of the immune response obtained. The tested adjuvants yielded responses as were expected from previous studies. However, the antigen often has some modulatory effect on top of what is expected for a given adjuvant. The choice of antigen can also influence the type of Th responses to some degree, thus the three selected antigens may provide a comprehensive coverage of various types of bias.

The proposed sets of harmonized protocols may be downloaded at: http://www.pharvat.eu/achievements. These are not intended to be in binding or optimal but propose to bridge various studies evaluating adjuvants. In the event routes or schedules other than those proposed here are employed, the inclusion of a bridging group (as per the proposed method) will facilitate directly a comparison with other studies and thus contribute to rational adjuvant selection. For instance, the QS21-Liposome formulations which induced high levels of immune responses with all antigens in all readouts would be a good candidate to compare the influence of varying experimental parameters.

Despite the efforts of harmonization, differences between experiments might still persist, and for example, efforts to further harmonize the animal work (sampling methods, animal handling, housing conditions, feeding, chronobiology, etc); could help at homogenizing further the final results of the different in vivo experiments. The mouse model is an advantageous model for many aspects (accessibility, cost, materials and tools available, etc.), but it has also some important limitations when the data obtained needs to be extrapolated to humans. The specificity of the murine immune system, the size and physiology of the animal, are fundamental issues when the effect of different adjuvants are compared as these criteria might impact on their adjuvant mechanism. New approaches are currently being developed in order to allow a better predictability of the response in humans like humanized mice models [28, 29] which may allow increase the value of the mouse model as a predictive tool of vaccine efficacy.

## Conclusions

The main output of this study was to demonstrate that standardizing protocols for adjuvant comparison is feasible and may allow the harmonized testing of vaccine adjuvants using reference materials. This is expected to facilitate direct comparison of studies performed with novel antigen-adjuvant combinations, and to contribute to more rationale adjuvant selection for vaccine researchers and developers.

The data presented here can be used as guidance and reference when designing adjuvant comparisons, and as potential tool for the rational selection of adjuvants for further development of experimental vaccines. Adjuvant developers are encouraged to follow this harmonization initiative or other initiatives [30]. Obviously, alternative administration schedules, other routes of immunization and other volumes of administration are possible. The protocols can be extended to other adjuvants and other antigens, but it remains essential that all antigen/adjuvant and control formulations are tested under exactly the same conditions and compared to results obtained with the original harmonized protocol. It should also be noted that the reference formulations are not suited to all routes of administration, for example the intranasal route.

The antigens AMA1 and Ag85A selected in this study can respectively be obtained through BPRC (Rijswijk, The Netherlands) and Lionex GmbH (Braunschweig, Germany) upon request. The ELISA reference sera can be obtained from BPRC upon request. The adjuvants selected in this study can be obtained through the Vaccine Formulation Laboratory (University of Lausanne, Switzerland) upon request.

## Abbreviations

Ag85A: Antigen 85A from mycobacterium tuberculosis
AlOH: Aluminium oxyhydroxide
AMA1: Apical membrane antigen 1
AU: Arbitrary unit
BPRC: Biomedical primate research centre
BSA: Bovine serum albumin
DOPC: Dioleoyl phosphatidyl choline
EEC: European union regulations
ELISA: Enzyme-linked immunosorbent assay
ELISPOT: Enzyme-linked immunosorbent spot
EPI: Expanded programme on immunisations
FCS: Fetal calf serum
GMP: Good manufacturing practices
HBsAg: Hepatitis B surface antigen
HIV: Human immunodeficiency virus
i.m.: intramuscularly
IFN-γ: Interferon-gamma
IgG: Immunoglobuline g
IgGt: Total Immunoglobuline g
IL-5: Interleukine-5
IP: Intellectual property
KW: Kruskal wallis
MAVF: Modern adjuvant / vaccine formulation
mBCA: Micro-bicinchoninic acid
MW test: Mann Whitney-U test
OD: Optical density
PBS: Phosphate buffered saline
PBS-T: Phosphate buffered saline with tween-20
pH: Hydrogen potential
PMA-Iono: Phorbol-myristate-acetate ionomycin
QS21: Purified saponin from *quillaja saponaria molina*
RPMI: Roswell park memorial institute
RT: Room temperature
SDS-PAGE: Sodium dodecyl sulfate polyacrylamide gel electrophoresis
SWE: Squalene oil in water emulsion
TMB: Tetramethylbenzidine
VFL: Vaccine formulation laboratory
WHO: World Health Organization
WSR-test: Wilcoxon signed rank test
